# Psychosocial correlates of parents’ willingness to vaccinate their children against COVID-19

**DOI:** 10.1371/journal.pone.0305877

**Published:** 2024-06-24

**Authors:** Hyunmin Yu, Stephen Bonett, Ufuoma Oyiborhoro, Subhash Aryal, Andrew Kim, Melanie L. Kornides, John B. Jemmott, Karen Glanz, Antonia M. Villarruel, José A. Bauermeister

**Affiliations:** 1 School of Nursing, University of Pennsylvania, Philadelphia, Pennsylvania, United States of America; 2 School of Nursing, Johns Hopkins University, Baltimore, Maryland, United States of America; 3 Annenberg School for Communication, University of Pennsylvania, Philadelphia, Pennsylvania, United States of America; 4 Perelman School of Medicine, University of Pennsylvania, Philadelphia, Pennsylvania, United States of America; Qazvin University of Medical Sciences, ISLAMIC REPUBLIC OF IRAN

## Abstract

**Background:**

Public health guidance recommended that children who are 6 months or older be vaccinated against COVID-19 in June of 2022. In the U.S., 56% of children under 17 had not received the COVID-19 vaccination in 2023. We examine parents’ willingness to vaccinate their children against COVID-19 using the theory of planned behavior in order to design effective strategies to promote vaccine uptake.

**Methods:**

The Philadelphia Community Engagement Alliance is part of an NIH community-engaged consortium focused on addressing COVID-19 disparities across the U.S. We surveyed 1,008 Philadelphia parents (mean age 36.86, SD 6.55; 42.3% racial/ethnic minorities) between September 2021 and February 2022, a period when guidance for child vaccination was anticipated. Structural Equation Modeling analysis examined associations between parental willingness and vaccine-related attitudes, norms, and perceived control. Covariates included parents’ COVID-19 vaccination status, race/ethnicity, gender, and survey completion post-CDC pediatric COVID-19 vaccination guidelines. Subgroup analyses by race/ethnicity and gender were conducted.

**Results:**

Our model demonstrated good fit (χ2 = 907.37, df = 419, *p*<0.001; comparative fit index [CFI] = 0.951; non-normed fit index [NNFI] = 0.946; root mean square error of approximation [RMSEA] = 0.034 with 95% CI = 0.030–0.038). Attitudes ($\hat{\beta }$ = 0.447, *p*<0.001) and subjective norms ($\hat{\beta }$ = 0.309, *p* = 0.002) were predictors of intention. Racial/ethnic minority parents exhibited weaker vaccination intentions ($\hat{\beta }$ = -0.053, *p* = 0.028) than non-Hispanic White parents.

**Conclusions:**

Parents’ attitudes and norms influence their vaccination intentions. Despite the survey predating widespread child vaccine availability, findings are pertinent given the need to increase and sustain pediatric vaccinations against COVID-19. Interventions promoting positive vaccine attitudes and prosocial norms are warranted. Tailored interventions and diverse communication strategies for parental subgroups may be useful to ensure comprehensive and effective vaccination initiatives.

## Introduction

The novel coronavirus disease 2019 (COVID-19) pandemic resulted in substantial human loss, exacerbated socio-economic inequities, and highlighted health system disparities, impacting communities globally [1–3]. Since the onset of the COVID-19 pandemic, public health authorities world-wide instituted an array of wide-scale measures to prevent the spread of the virus, including social distancing, hand hygiene, mask-wearing, travel restrictions, and quarantine protocols [4]. Central to these containment efforts was vaccination [5, 6]. After the World Health Organization (WHO) urged the pursuit of COVID-19 vaccine research and development, remarkable global vaccine research efforts led to the expeditious development and approval of several COVID-19 vaccines, including those developed by Moderna, Pfizer/BioNTech, and AstraZeneca [7].

Despite these significant strides and the vital role of vaccination in promoting wellbeing and quality of life during the COVID-19 pandemic [8, 9], vaccine hesitancy and refusal emerged as widespread challenges, posing barriers to achieving herd immunity [10–12] and influencing decision-making processes [13, 14]. Notably, in the U.S., as of May 2023, 56% of children under the age of 17 had not yet received the COVID-19 vaccination [15]. While studies have examined various factors associated with parental intentions to vaccinate their children against COVID-19, including social norms, sociodemographic characteristics, and vaccination-related attitudes [16, 17], understanding the theory-informed factors that influence parents’ intentions regarding the COVID-19 vaccine for their children is crucial in ensuring widespread vaccine acceptance and optimal protection against infection and severe disease.

The Theory of Planned Behavior (TPB) [18, 19] provides a robust framework to examine and predict human behavior, particularly in health-related decision-making. TPB posits and research confirms [20, 21] that behavioral intentions strongly predict implemented behavior, especially in the context of reasoned and goal-directed actions. According to the TPB, behavioral intentions are influenced by three key constructs: (1) the individual’s favorable or unfavorable perception and evaluation of a particular behavior (attitudes toward behavior), (2) perceptions of social approval to perform or not perform the behavior (subjective norms), and (3) the perception of the ease or difficulty of executing the behavior (perceived behavioral control). These factors collectively shape an individual’s intention to engage in a specific behavior, and thus are important determinants of actual behavior [19].

The TPB has demonstrated its effectiveness as a reliable predictor of both COVID-19 vaccine intentions and COVID-19 vaccination, as evidenced by multiple studies conducted across various countries and populations [22]. Some studies applied the TPB to investigate people’s intentions to receive future hypothetical vaccines prior to the release of the COVID-19 vaccines [23–26]. After the COVID-19 vaccines became available, additional studies have investigated the relationship between TPB and people’s intentions to receive these vaccines [27–31].

Table 1 summarizes findings from prior US-based studies that utilized the TPB to investigate intentions to vaccinate against COVID-19. All centered around adult populations, with one emphasizing older adults aged 60 or above [32], and another highlighting young adults, specifically college students [26]. Only one of the eight studies we identified reported that all three core TPB constructs (attitudes, subjective norms, and perceived behavioral control) were predictors of COVID-19 vaccination intention [33]. Four studies found that attitudes and subjective norms were significant predictors of COVID-19 vaccine intention, while perceived behavioral control was not significantly associated with intention [23, 32, 34, 35]. Another study [36] found attitudes and perceived behavioral control to be predictors and subjective norms to be unrelated. A different study [37] indicated that attitudes and subjective norms were predictive, but it did not include perceived behavioral control in the analyses. A study focusing on college students [26] reported that only subjective norms were found to be significant, while attitudes and perceived behavioral control were not.

**Table 1:** US-based studies applying the TPB to examine COVID-19 vaccination intention.

Authors (Years)	Study Period	Region	Population	*N*	Included TPB Constructs	Key Findings
Berg and Lin (2021) [34]	Before vaccines were available.	Not reported	Adult (Age: 18~81, Mean 46.32 ± 16.44)	337	ATT, SN, PBC	ATT and SN were found to be predictors of COVID-19 vaccination intention, while PBC did not show significant predictive influence.
Callow and Callow (2021) [32]	Before vaccines were available.	Delaware, Maryland, Virginia, Washington, D.C.	Older adults (Age: ≥ 60)	583	ATT, SN, PBC	ATT and SN were found to be associated with COVID-19 vaccination intention, while PBC did not show a significant impact on intention.
Chu and Liu (2021) [23]	Before vaccines were available.	Not reported	Adults (Mean age: 46.01 ± 16.17)	934	ATT, SN, PBC	ATT and SN were found to be associated with COVID-19 vaccination intention, while PBC did not show a significant impact on intention.
Ekinci et al. (2022) [37]	Data collection period was unclear.	Not reported	Adults (Age: ≥ 18)	1008	ATT, SN	ATT and SN were found to be predictors of COVID-19 vaccination intention.
Guidry et al. (2021) [35]	Before vaccines were available.	Not reported	Adults (Mean age: 45.9 ± 17.15)	788	ATT, SN, PBC	ATT and SN were found to be predictors of COVID-19 vaccination intention, while PBC did not show significant predictive influence.
Hagger and Hamilton (2022) [33]	After vaccines were available.	Not reported	Adults (Mean age: 52.14 ± 14.55)	479	ATT, SN, PBC	ATT, SN and PBC were found to be associated with COVID-19 vaccination intention.
Hayashi et al. (2022) [36]	After vaccines were available.	Not reported	Adults (Age: ≥ 18)	172	ATT, SN, PBC	ATT and PBC were found to be predictors of COVID-19 vaccination intention, while SN did not show significant predictive influence.
Reyes et al. (2023) [26]	Before vaccines were available.	New England region	Young adults (Age: 18 ~30, Mean 21.81 ± 3.04)	170	ATT, SN, PBC	SN was identified as a significant factor associated with COVID-19 vaccination intention, while ATT and PBC did not exhibit a significant impact on intention.

Abbreviation: ATT = attitudes, SN = subjective norms, PBC = perceived behavioral control

The varied findings among these studies highlight the complexity of factors influencing vaccination intention and underscore the importance of understanding these factors in different populations and contexts. No previous studies have proposed a TPB-based model to examine parents’ intention to have their children vaccinated. Addressing this research gap is crucial for gaining a comprehensive and essential understanding of the dynamics behind family vaccination decision-making.

Conducting a study that examines parents’ intention to have their children vaccinated is greatly significant for several reasons. First, a TPB-informed model would enable researchers to understand the factors that influence COVID-19 vaccination decision-making within families through the exploration of parental attitudes. Second, understanding the intentions of parents can inform the design of communication campaigns, educational initiatives, and interventions aimed at promoting vaccine acceptance and improving vaccination rates across various age groups. Moreover, parental attitudes and decisions profoundly impact children’s healthcare choices and outcomes [38, 39]. Therefore, comprehending parents’ intentions is essential in shaping vaccination among the younger population.

Our research aims to use TPB as the foundational framework to identify the underlying factors influencing parents’ intentions to have their child receive the COVID-19 vaccine, including attitudes, perceived behavioral control, and subjective norms, and to understand how these constructs influence their ultimate decision-making.

**Fig 1:**
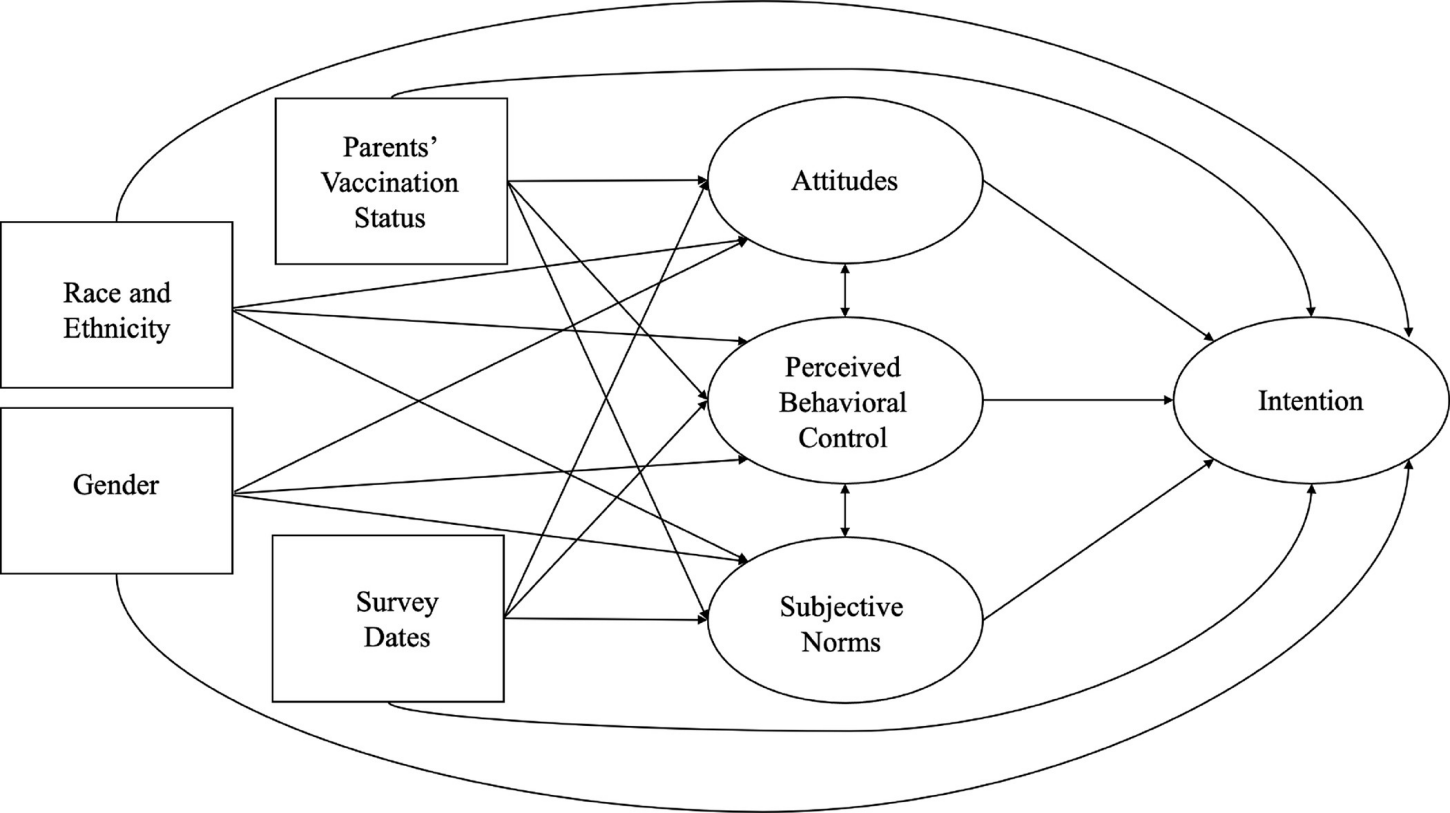
Parents’ vaccination intentions as informed by the theory of planned behavior.

Our hypotheses are:

1. Consistent with the TPB, we hypothesized that there is a direct positive association between COVID-19 vaccination intention and the following constructs within parent populations: (a) attitudes toward COVID-19 vaccination; (b) subjective norms; and (c) perceived behavioral control (Fig 1). The hypothesized TPB model (Fig 1) includes four latent variables: (1) intention, (2) attitudes, (3) perceived behavioral control (PBC), and (4) subjective norms (SN) and four observed variables: parental vaccination status, survey completion dates, race/ethnicity and gender.
2. Given reported differences in vaccine-related attitudes and uptake by race/ethnicity [40–42], we hypothesized that there is a difference in these associations between non-Hispanic White parents and parents from racial and ethnic minority backgrounds.
3. Give emergent data regarding differences by parental gender in COVID-19 vaccination [43–45], we further hypothesized that there would be a difference in these associations between male parents and female parents.

## Methods

### Procedure

This cross-sectional study is a part of the broader Philadelphia CEAL (Community Engagement Alliance) study, which aimed to mitigate disparities in COVID-19 testing, vaccine uptake, and clinical trial participation among communities disproportionately affected by the pandemic in the city of Philadelphia. Initially, 8,153 people were recruited via online and community-based outreach to participate in a baseline self-administered screener and confidential survey from September 7^th^ 2021 to February 14^th^ 2022. Eligibility to participate in the survey required participants to reside in Philadelphia and be at least 13 years of age. A total of 3,936 participants were initially deemed valid and eligible through a real-time fraud detection protocol developed for this study. After an additional post-hoc fraud detection process, the number of valid participants was further narrowed down to 2,870. Detailed information regarding the fraud detection process used for this study has been published elsewhere [46–48].

For this study, we extracted the subset of participants from the original dataset that identified as parents. The parent sample consisted of 1,309 participants, each of whom had at least one child ≤ 17, irrespective of the participant’s age. All study procedures were approved by the University of Pennsylvania Institutional Review Board (#848650), and written informed consents were acquired from all participants.

### Measures

#### TPB constructs

The questionnaire about COVID-19 vaccines for parent participants were developed based on the TPB [19]. It encompassed the original constructs of the TPB, which consisted of (1) intention, (2) attitudes, (3) perceived behavioral control, and (4) subjective norms. Multiple items were utilized to measure each TPB construct. For the questionnaire, positively coded items were employed, encompassing 9 attitudes items, 7 subjective norms items, and 9 perceived behavioral control items. The wording of these items across the three constructs can be found in Table 2. All responses were recorded on a five-point Likert scale, ranging from ‘1 = very unlikely’ to ‘5 = very likely.’ Higher scores for each construct indicated more positive attitudes, greater degree of positive subjective norms, and higher perceived behavioral, respectively, among the participants.

**Table 2:** The Theory of Planned Behavior (TPB) measurement model.

Latent Factors	*N*	Items	Mean	SD	Standardized Factor Loadings
Attitude (α = 0.87)	1007	Receiving the COVID-19 vaccine would/does protect my child from getting COVID-19.	3.9	0.99	0.660
	1008	If my child receives a COVID-19 vaccine, they could go to school in person.	3.9	1.01	0.572
	1006	If my child receives a COVID-19 vaccine, they could/can safely participate in school, group, and sports activities.	3.9	0.98	0.617
	1006	If my child receives a COVID-19 vaccine, they could/can travel safely.	3.9	0.96	0.608
	1006	If my child receives a COVID-19 vaccine, I would be/am less worried about them getting COVID-19.	3.9	1.01	0.689
	1006	My child receiving a COVID-19 vaccine would/does help protect our community from COVID-19.	4.0	0.99	0.679
	1007	My child receiving the COVID-19 vaccine would/does make my child safe around other people.	3.9	0.98	0.677
	1006	My child receiving a COVID-19 vaccine would/does help protect other students and teachers in their school from COVID-19.	3.9	0.99	0.680
	1006	My child receiving a COVID-19 vaccine would/does protect others in my family from getting COVID-19.	3.9	0.97	0.667
Subjective Norm (α = 0.77)	998	Most people who are important to me would approve of my child receiving a COVID-19 vaccine.	4.0	0.96	0.603
	978	My spouse/partner would approve of my child receiving a COVID-19 vaccine.	4.0	1.04	0.635
	995	My family would approve of my child receiving a COVID-19 vaccine.	4.0	1.00	0.630
	994	My friends would approve of my child receiving a COVID-19 vaccine.	4.0	0.97	0.626
	999	My doctor would approve of my child receiving a COVID-19 vaccine.	4.1	0.93	0.516
	842	My pastor or other religious leader would approve of my child receiving a COVID-19 vaccine.	3.9	0.90	0.519
	959	My child would approve of receiving a COVID-19 vaccine.	3.9	1.04	0.602
Perceived Behavioral Control (α = 0.87)	1007	I am sure I can have my child receive a COVID-19 vaccine, even if I have many problems in my life.	3.9	0.95	0.650
	1007	I am sure I can have my child receive a COVID-19 vaccine, even if I am very busy.	3.9	0.97	0.663
	1007	I am sure I can have my child receive a COVID-19 vaccine, even if it is hard to find a place that offers the vaccine.	3.9	0.96	0.676
	1006	I am sure I can have my child receive a COVID-19 vaccine, even if it is offered at inconvenient times.	3.9	0.98	0.669
	1007	I am sure I can have my child receive a COVID-19 vaccine, even if it is hard to make an appointment.	3.9	0.98	0.663
	1006	I am sure I can have my child receive a COVID-19 vaccine, even my child is against receiving the vaccine.	3.9	1.00	0.587
	1007	I am sure I can have my child receive a COVID-19 vaccine, even if I have to take time off from work or school to take them to be vaccinated.	3.9	0.98	0.677
	1007	I am sure I can have my child receive a COVID-19 vaccine, even if the place that offers the vaccine is far from my home.	3.9	1.02	0.651
	1008	I am sure I can have my child receive a COVID-19 vaccine, even if the waiting time is very long.	3.9	0.98	0.681
Intention	1008	I am willing to have my child receive a COVID-19 vaccine.	3.9	1.05	Not applicable

#### Vaccination intent

The intention of parent participants to have their child vaccinated was evaluated using a single item: “I am willing to have my child receive a COVID-19 vaccine.” Parents provided their answers on a scale ranging from ‘1 = strongly disagree’ to ‘5 = strongly agree,’ with additional options for ‘2 = somewhat disagree,’ ‘3 = neither agree nor disagree,’ and ‘4 = somewhat agree.’

#### Parent vaccination history

Parents’ prior COVID-19 vaccination status was measured through one item: ‘Have you received at least one dose of the COVID-19 vaccine?’ Participants selected from several response options, including ‘1 = yes, got one-dose vaccine,’ ‘2 = yes, got first dose of two-dose vaccine,’ ‘3 = yes, got both doses of a two-dose vaccine,’ ‘4 = no, have not received the vaccine,’ ‘5 = don’t know,’ ‘6 = prefer not to answer,’ and ‘7 = yes, got more than two doses of a vaccine (e.g., an additional dose or booster).’ The responses were modified into five categories to enhance clarity, merging ‘1’ and ‘3’ as ‘yes, got full primary series,’ and combining ‘5’ and ‘6’ as ‘don’t know/prefer not to answer.’

#### Pediatric vaccine recommendation

Taking into account the Centers for Disease Control and Prevention’s (CDC) initial guideline, which recommended pediatric COVID-19 vaccines for children on November 2, 2021 [49], the completion dates of parents’ surveys were dichotomized into ‘0 = on or before 11/2/2021, before recommendation’ and ‘1 = after 11/2/2021, after recommendation.’

### Statistical analysis

We conducted descriptive statistical analyses, encompassing frequency distributions and means, using R version 4.2.3. To examine the hypothesized TPB model, we employed Structural Equation Modeling (SEM) through the Lavaan [50, 51]. We employed a two-stage modeling approach [52]. In the initial stage, Confirmatory Factor Analysis (CFA) was conducted to test the factorial validity of the latent constructs and the adequacy of the measurement model. The three latent constructs, namely attitudes, perceived behavioral control, and subjective norms, were included in this stage. The standardized correlation coefficients were assessed to determine if they fell below the cut-off point of 0.85 [53]. To identify potential sources of misfit, modification indices were examined, providing a foundation for re-specifying the measurement model as needed.

After specifying the measurement model, the next step involved performing SEM to assess whether the hypothesized TPB model demonstrated acceptable fit to the data and to estimate the effects of relationships within the model. The SEM model comprised three latent variables, with parental vaccination status, binary survey completion dates before and after the CDC’s recommendation for the pediatric vaccine, gender, race and ethnicity included as observed variables. Several statistical parameters were employed to evaluate the model’s fit to the data, including the chi-square (χ^2^) test, comparative fit index (CFI), non-normed fit index (NNFI), root mean square error of approximation (RMSEA), and standardized root mean square residual (SRMR) [54]. A statistically non-significant result for the chi-square test (*p* > 0.05) would indicate a good fit for the model. However, it is noted that the chi-square test’s significance is highly influenced by sample size, large samples can lead to significant *p*-values even with minor misspecifications [55]. With this, focus was placed on the remaining fit indices. In accordance with established guidelines [54, 56], good fit is indicated by CFI and NNFI values exceeding 0.90 or 0.95, an RMSEA value below 0.06, and an SRMR value under 0.05. These fit indices served as key indicators to evaluate the adequacy of the hypothesized TPB model with the collected data.

After establishing the SEM, we conducted a subgroup analysis to compare a SEM model for parents from racial and ethnic minority backgrounds with a model for non-Hispanic White parents. Additionally, we compared a SEM model for cisgender male parents with a model for cisgender female parents. These analyses were performed based upon previous research evidence indicating differences in vaccination intentions based on race, ethnicity, and gender [57–59].

We excluded 301 participants who did not respond to any survey items from the initial 1,309, resulting in 1,008 participants included in the CFA and SEM analyses. For participants with partially missing survey data, we utilized multiple imputations by chained equations [60], and the imputed datasets using Rubin’s rules [61] with a common set of *m* = 5 imputations. This approach facilitated data retention in multivariable models, enhancing the robustness and reliability of the analysis. After completing the imputation process, a binary indicator variable was generated, denoting ‘0 = not imputed/no missing’ and ‘1 = imputed.’ This variable was subsequently employed as a control variable in the SEM analysis. Its inclusion as a control variable allowed for the examination and potential adjustment of any imputation-related effects on the SEM results, contributing to the overall rigor and validity of the study’s findings. We additionally conducted a sensitivity analysis, affirming the consistency of results between our observed data and imputed data for our overall structural model. Last, we enhanced our model by introducing bootstrap standard errors with 1,000 replicates. This technique adds an additional layer of reliability to our analysis by accounting for potential sampling variability.

## Results

Our final study sample consisted of 1008 Philadelphia parents (Table 3). Their average age of was 36.86 (SD 6.55). About 57.7% of the parent participants identified as non-Hispanic White, 65.1% identified as women, and 92.8% identified as heterosexual. Parents who received more than one COVID-19 vaccine accounted for 97.2%, while those who did not receive any COVID-19 vaccine constituted 2.4%, and 0.4% chose not to share their vaccination status. Participants who completed surveys on or before the date the vaccine was recommended by the CDC for children (11/2/2021) versus those who completed them after did not exhibit statistically significant differences in their attitudes (M 4.11, SD 1.00; M 3.92, SD 0.68; *p* = 0.419), subjective norms (M 4.22, SD 0.85; M 3.94, SD 0.58; *p* = 0.197), perceived behavioral control (M 4.24, SD 0.85; M 3.89, SD 0.68; *p* = 0.087) and intention (M 4.00, SD 1.55; M 3.86, SD 1.04; *p* = 0.679).

**Table 3:** Demographic characteristics for the participating parents.

	Parent (n = 1008)
**Age (mean (SD))**	36.86 (6.55)
**Race and Ethnicity (%)**	
Hispanic/Latinx	79 (7.8)
Non-Hispanic Multiracial/Other	20 (2.0)
Non-Hispanic American Indian or Alaska Native	4 (0.4)
Non-Hispanic Asian	39 (3.9)
Non-Hispanic Black or African American	279 (27.7)
Non-Hispanic Native Hawaiian/Pacific Islander	5 (0.5)
Non-Hispanic White	582 (57.7)
**Gender (%)**	
Woman	656 (65.1)
Man	343 (34.0)
Transgender or gender diverse	5 (0.5)
Prefer not to answer	4 (0.4)
**Sexual Orientation (%)**	
Straight (i.e., not gay, lesbian or bisexual)	935 (92.8)
Bisexual	37 (3.7)
Gay	5 (0.5)
Lesbian	11 (1.1)
Other	13 (1.3)
Prefer not to answer	7 (0.7)
**Parents’ Vaccination Status (%)**	
Yes, got full primary series	891 (88.4)
Yes, got more than two doses of a vaccine (e.g., an additional dose or booster)	6 (0.6)
Yes, got first dose of two-dose vaccine	83 (8.2)
No, have not gotten the vaccine	24 (2.4)
Don’t know/Prefer not to answer	4 (0.4)
**Survey Completion Date (%)**	
Completed on or before 11/2/2021	21 (2.1)
Completed on or after 11/3/2021	987 (97.9)

### Measurement model

In the full measurement model for parents the standardized factor loadings ranged from 0.461 to 0.689 and were all significant (*p* < 0.001; Table 2). The standardized correlation coefficients ranged from 0.768 to 0.864 (S1 Appendix). All factor correlations with one exception remained below the cut-off point of 0.85. These findings collectively underscore the model’s overall satisfactory level of discriminant validity across the latent constructs.

The hypothesized three-factor measurement model showed a good fit, as evidenced by the fit indices (χ2 = 544.231; df = 272, *p* < 0.001; CFI = 0.970; NNFI = 0.967; RMSEA = 0.032 with 95% CI = 0.027–0.037; SRMR = 0.029). Post-hoc modifications were unnecessary due to the good fit of the data to the model.

### Structural model

**Fig 2:**
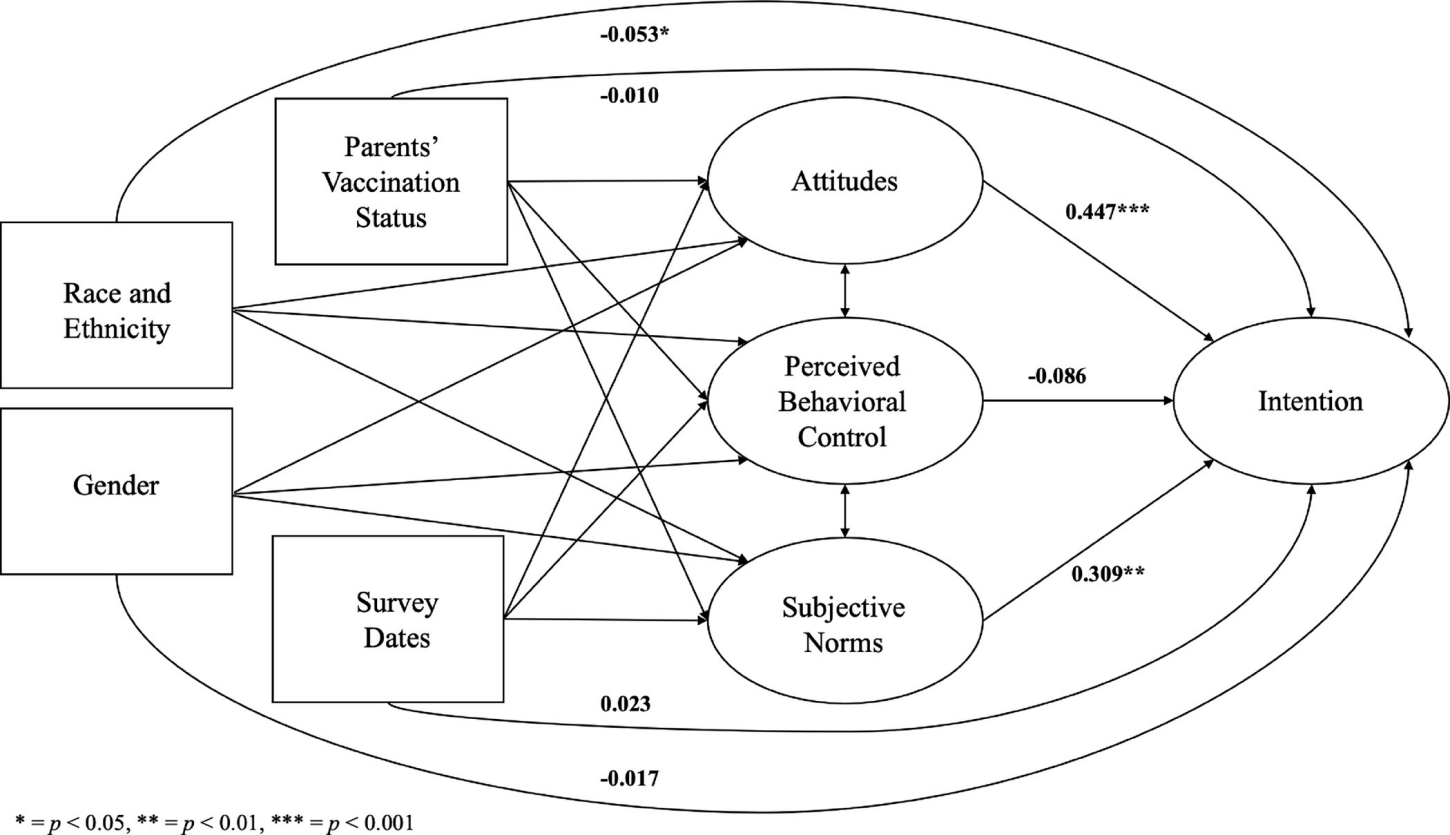
The overall structural equation model. * = *p* < 0.05, ** = *p* < 0.01, *** = *p* < 0.001.

The full structural model exhibited a good fit (χ2 = 907.370; df = 419, *p* < 0.001; CFI = 0.951; NNFI = 0.946; RMSEA = 0.034 with 95% CI = 0.030–0.038; SRMR = 0.050). Among the constructs, attitudes displayed the strongest predictive power on intention (standardized $\hat{\beta }$ = 0.447, *p* < 0.001), followed by subjective norms (standardized $\hat{\beta }$ = 0.309, *p* = 0.002) (Table 4). The effect of perceived behavioral control on intention was negative, albeit lacking statistical significance at the level of 0.05 (standardized $\hat{\beta }$ = -0.086, *p* = 0.171). Among observed variables, race and ethnicity were statistically significantly associated with intention (standardized $\hat{\beta }$ = -0.053, *p* = 0.028, indicating parents from racial/ethnic minority backgrounds expressed a weaker intention to vaccinate their children than non-Hispanic White parents. Parents’ vaccination status (standardized $\hat{\beta }$ = -0.010, *p* = 0.727), the survey completion dates (standardized $\hat{\beta }$ = 0.023, *p* = 0.361) and gender (standardized $\hat{\beta }$ = -0.017, *p* = 0.486) were not significantly associated with intention (Fig 2).

**Table 4:** Estimated regression coefficients for the structural equation model.

Effects	Unstandardized $\hat{\beta }$	95% CI	Standardized $\hat{\beta }$	95% CI
Overall (n = 1008)				
Attitude	0.712*	0.430 to 1.001	0.447*	0.267 to 0.627
Subjective norm	0.551*	0.220 to 0.897	0.309*	0.115 to 0.503
Perceived behavioral control	-0.145	-0.357 to 0.051	-0.086	-0.207 to 0.036
Non-Hispanic White (n = 582)				
Attitude	0.361	-0.055 to 0.880	0.283	-0.053 to 0.620
Subjective norm	0.405*	0.033 to 0.789	0.318*	0.036 to 0.600
Perceived behavioral control	0.050	-0.273 to 0.346	0.039	-0.198 to 0.276
Race and ethnic minority (n = 426)				
Attitude	0.745*	0.400 to 1.114	0.530*	0.291 to 0.768
Subjective norm	0.422*	0.024 to 0.841	0.299*	0.017 to 0.582
Perceived behavioral control	-0.201	-0.422 to 0.022	-0.143	-0.297 to 0.011
Male (n = 343)				
Attitude	0.697	-0.104 to 1.516	0.412	-0.060 to 0.884
Subjective norm	0.443	-0.464 to 1.355	0.236	-0.237 to 0.709
Perceived behavioral control	-0.047	-0.511 to 0.415	-0.028	-0.308 to 0.251
Female (n = 656)				
Attitude	0.715*	0.391 to 1.082	0.455*	0.238 to 0.671
Subjective norm	0.582*	0.194 to 1.030	0.331*	0.097 to 0.564
Perceived behavioral control	-0.181	-0.461 to 0.048	-0.104	-0.248 to 0.040

* = *p* < 0.05

*Control variables included in the model*: parents’ prior COVID-19 vaccination status, survey completion dates (before and after the CDC’s pediatric vaccine recommendation), and imputation status

### Subgroup analyses

**Fig 3:**
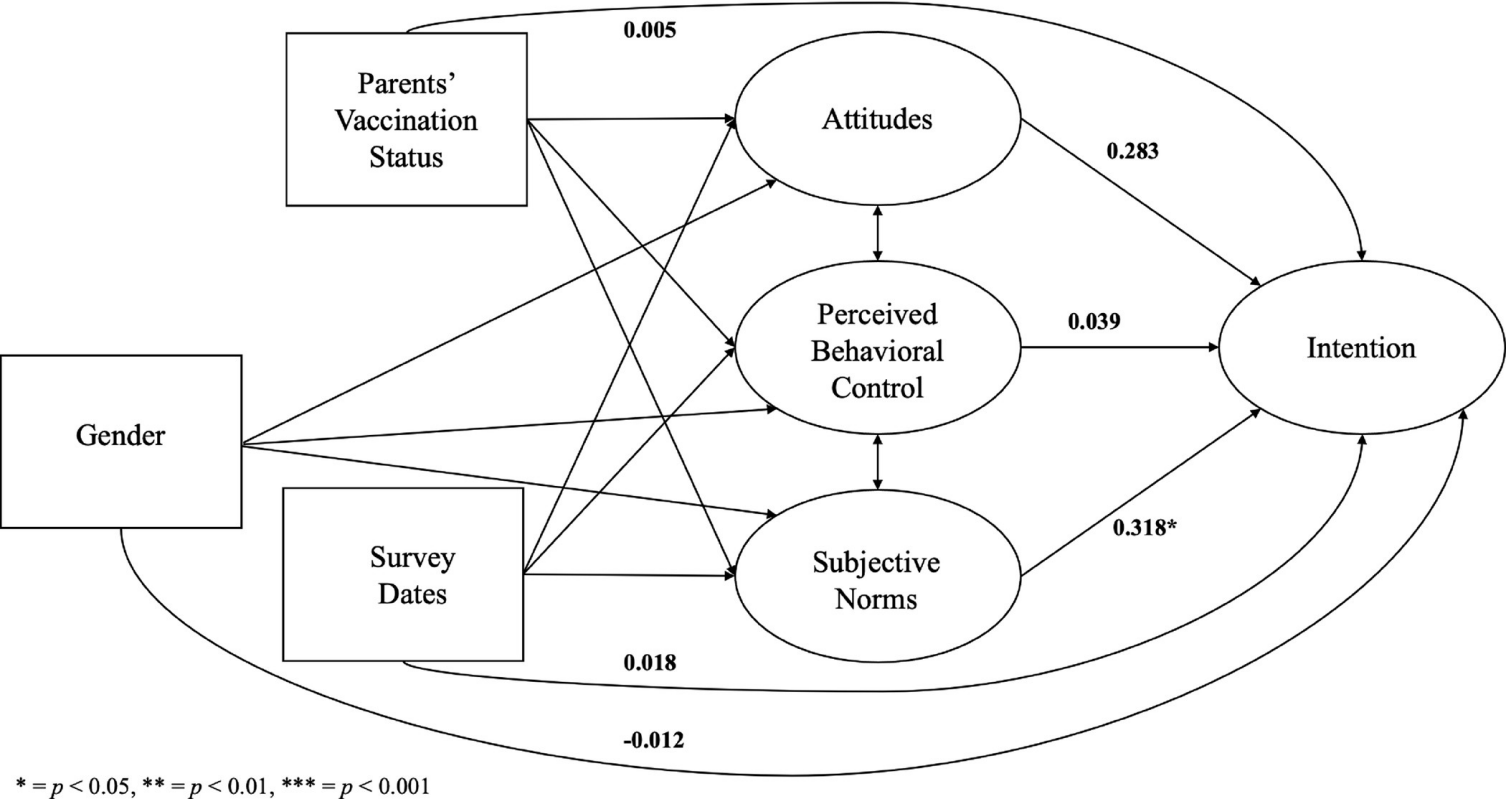
The structural equation model for racial and ethnic minority parents. * = *p* < 0.05, ** = *p* < 0.01, *** = *p* < 0.001.

**Fig 4:**
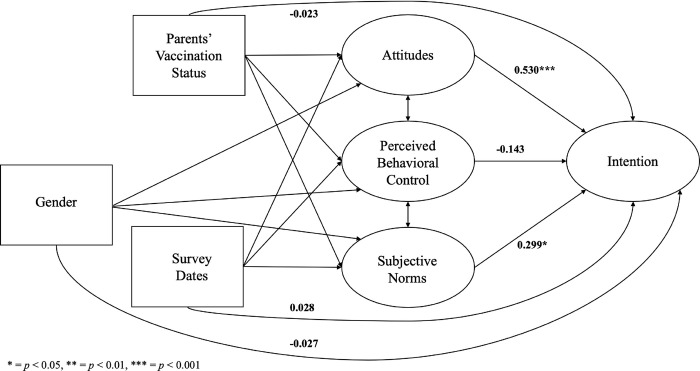
The structural equation model for Non-Hispanic White parents. * = *p* < 0.05, ** = *p* < 0.01, *** = *p* < 0.001.

**Fig 5:**
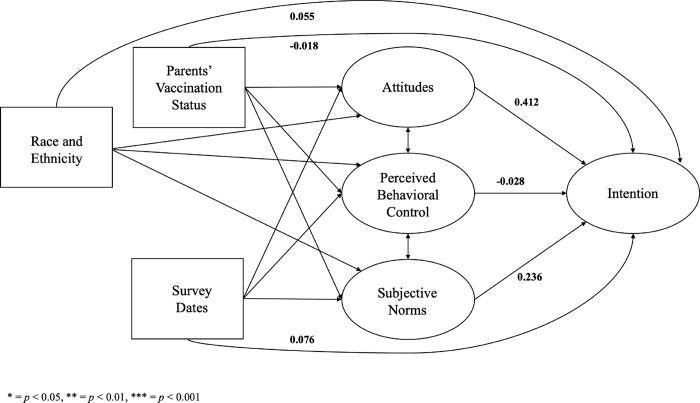
The structural equation model for male parents. * = *p* < 0.05, ** = *p* < 0.01, *** = *p* < 0.001.

**Fig 6:**
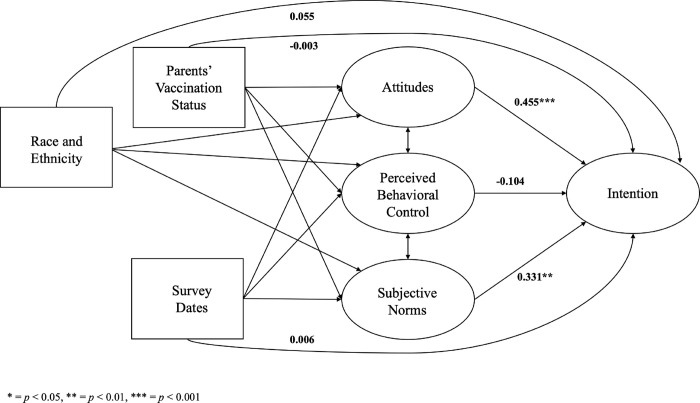
The structural equation model for female parents. * = *p* < 0.05, ** = *p* < 0.01, *** = *p* < 0.001.

The hypothesized effects exhibited variations across racial and ethnic subgroups (Table 4). Among parents from racial and ethnic minority backgrounds, attitudes (standardized $\hat{\beta }$ = 0.530, *p* < 0.001) and subjective norms (standardized $\hat{\beta }$ = 0.299, *p* = 0.040) had a significant impact on intention to have their children vaccinated (Fig 3). However, among non-Hispanic White parents, subjective norms alone were found to have a significant influence on intention (standardized $\hat{\beta }$ = 0.318, *p* = 0.031), while attitudes did not demonstrate a statistically significant effect (standardized $\hat{\beta }$ = 0.283, *p* = 0.100) (Fig 4). Furthermore, the effects varied across cisgender subgroups. Among male parents, none of the TPB constructs were significantly associated with intention (Fig 5). However, among female parents, attitudes (standardized $\hat{\beta }$ = 0.455, *p* < 0.001) and subjective norms (standardized $\hat{\beta }$ = 0.331, *p* = 0.006) had a significant impact on intention (Fig 6).

## Discussion

In this study we used TPB, a well-established theoretical framework, to predict parents’ intention to vaccinate their children against COVID-19. The theory holds that an attitude toward the behavior, the subjective norms regarding the behavior, and perceived behavioral control have direct and independent effects on intention. Structural equation modeling revealed that attitudes and subjective norms predicted vaccine intention, consistent with TPB. Parents who favorably evaluated vaccinating their children and those who perceived support for vaccinating the children among their important referents expressed a greater willingness to vaccinate their children against COVID-19. However, contrary to the theory, perceived behavioral control, beliefs about how easy or hard it would be to vaccinate their children against COVID-19, was unrelated to their intention.

A basic tenet of TPB is that the relative predictive power of attitudes, subjective norms, and perceived behavioral control varies depending on the behavior and the population in question [19]. Our findings suggest that attitudes and subjective norms may be more influential than perceived behavioral control on the intention of parents to vaccinate their children against COVID-19. The inability of perceived behavioral control to predict intention may mean that the parents in our population did not differ much in whether they had the necessary resources and opportunities to acquire the vaccine for their children. Although this explanation is only conjecture, our finding that perceived behavioral control is unrelated to vaccine intention has been observed in other studies. Indeed, most studies have found that attitudes and subjective norms affect vaccine intentions more than perceived behavioral control [23, 32, 34, 35]. This study is the first to investigate parents’ intentions to vaccinate their children against COVID-19. It suggests that the previously observed relationship in studies focused on adult populations, where perceived behavioral control is unrelated to vaccine intentions for adults themselves, also applies to parents’ intentions regarding COVID-19 vaccinations for their children.

Our study further explored these intentions within the contexts of racial and ethnic backgrounds and gender, shedding light on how these factors significantly influence vaccination-related decision-making. Notably, our analysis revealed substantial variations in the effects of key TPB constructs across different racial and ethnic subgroups. Among parents from racial and ethnic minority backgrounds, attitudes and subjective norms exhibited significant positive associations with the intention to have their children vaccinated. These findings align with prior research underscoring the importance of attitudes and social norms in shaping vaccination behavior [58, 59, 62]. The robust relationship between positive attitudes and vaccination intention highlights the significance of individuals’ overall favorable perceptions of vaccination for their children. Additionally, the role of subjective norms underscores the influence of social networks and cultural contexts on vaccination decisions. In contrast, for non-Hispanic White parents, social norms appeared to play a more prominent role in shaping their intentions to vaccinate their children. The significance of subjective norms in this subgroup emphasizes the importance of understanding community-level influences and peer interactions in vaccination decision-making processes [63, 64].

Furthermore, our findings demonstrated variations across cisgender subgroups. While previous studies have reported higher vaccination intentions among males compared to females [57, 58, 65], these findings were typically related to self-vaccination rather than vaccination decisions for their children. Among male parents in our study, none of the TPB constructs displayed a statistically significant association with vaccination intention. This suggests that factors beyond the scope of TPB measurements may be influencing vaccination decisions in this subgroup. In contrast, among female parents, attitudes and subjective norms emerged as significant predictors of vaccination intention, aligning with the overall findings.

The attitudes and subjective norms associated with intentions in the present study are modifiable. Decisions about which variables to target with an intervention should be based on empirical evidence rather than broad generalizations. Thus, for those who would intervene to increase parents’ intention to vaccinate their children against COVID-19, the present results suggest that strategies should be utilized to instill positive attitudes among parents toward vaccinating their children and supportive normative beliefs regarding their significant referents. In this connection, strategies need to be developed to increase positive evaluations of vaccination for children. Social network interventions may also be an effective strategy to change parents’ subjective norms about vaccinating their children.

Several factors must be weighed when evaluating this study. A strength of the study is the use of TPB, a theory-based approach. Another strength is the large sample, including many minoritized individuals. However, the use of a convenience sample and substantial complete missingness for the TPB items may introduce selection bias, limiting the generalizability of the findings to all parents. Another potential limitation is the self-reported data, which may introduce response bias and social desirability bias. Participants may have provided responses that they perceived as socially acceptable or in line with societal norms, potentially affecting the accuracy of their reported attitudes and intentions regarding vaccination. Nonetheless, our study provides insight and context for the behavioral factors influencing parents’ intentions to have their children vaccinated among a sample of Philadelphia parents. Future research could delve deeper into the mechanisms underlying variations among different parental subpopulations and explore additional factors that may contribute to vaccination decision-making within specific subgroups. Moreover, ongoing efforts to address vaccine hesitancy and enhance vaccination rates should consider tailoring strategies to the unique characteristics and influences within these subpopulations.

## Conclusions

In conclusion, our study contributes to the growing body of literature on vaccination behavior by highlighting the differential influences of attitudes, subjective norms, and intentions regarding parents vaccinating their children across racial, ethnic, and gender subgroups. These findings underscore the need for targeted interventions and communication strategies that consider the unique factors shaping vaccination intentions among diverse populations.

There is a great need for theory-driven interventions to increase COVID-19 vaccine uptake, including interventions to encourage U.S. parents to vaccinate their children. Efforts to understand and change intentions in this population might be most successful if they focus on attitudes and subject norms, as the present research has highlighted. Future research must clarify why perceived behavioral control is unrelated to intention. Research along these lines would contribute to efforts to reduce the children’s risk of COVID-19 infection and, more generally, help curb the COVID-19 pandemic.

## Supporting information

S1 Appendix Standardized factor correlation coefficients. (DOCX)
