# Correction: Psychosocial correlates of parents’ willingness to vaccinate their children against COVID-19

**DOI:** 10.1371/journal.pone.0323661

**Published:** 2025-04-29

**Authors:** Hyunmin Yu, Stephen Bonett, Ufuoma Oyiborhoro, Subhash Aryal, Andrew Kim, Melanie L. Kornides, John B. Jemmott, Karen Glanz, Antonia M. Villarruel, José A. Bauermeister

There are errors in the Funding statement. The correct Funding statement is as follows: This research was, in part, funded by the National Institutes of Health (NIH) Agreement OT2HL158287. The views and conclusions contained in this document are those of the authors and should not be interpreted as representing the official policies, either expressed or implied, of the NIH.
