# Supplementary material for: Psychosocial correlates of parents’ willingness to vaccinate their children against COVID-19
Source: PLoS One. 2024 Jun 24;19(6):e0305877. doi: 10.1371/journal.pone.0305877 (PMC11195945; doi:10.1371/journal.pone.0305877)
Supplement: S1 Appendix — (DOCX) [file pone.0305877.s001.docx]

**S1 Appendix. Standardized Factor Correlation Coefficients**

| **Constructs** | **Correlation coefficients** |
| --- | --- |
| Attitude – Subjective norm | 0.864 |
| Attitude – Perceived behavioral control | 0.767 |
| Attitude – Intention | 0.654 |
| Subjective norm – Perceived behavioral control | 0.789 |
| Subjective norm – Intention | 0.633 |
| Perceived behavioral control – Intention | 0.504 |
